# Humanity’s urgent challenges need solutions from systems microbiology

**DOI:** 10.1128/msystems.00838-25

**Published:** 2025-07-01

**Authors:** Ashley Shade

**Affiliations:** 1 Universite Claude Bernard Lyon 1, CNRS, INRAE, VetAgro Sup, Laboratoire d'Ecologie Microbienne LEM, CNRS UMR5557, INRAE UMR1418, Villeurbanne, France

## EDITORIAL

It is with great excitement and sincere dedication that I assume the role of editor in chief of *mSystems*. Building on the momentum and reputation that *mSystems* has achieved in its first decade, we will continue the journal’s tradition of pushing boundaries, upholding scientific integrity, and fostering community.

I am humbled and a bit awestruck by this opportunity to lead, serve, and learn. I taste it with some bittersweetness because my stepping up means that our founding editor in chief, Jack Gilbert, is bowing out. Jack empowered each of us to take risks, do the right thing, and follow our best ideas to advance *mSystems*. He is already missed.

I have had the privilege of serving *mSystems* since its birth. Almost 10 years ago, Jack invited me to embark on an adventure: join the editorial board of a brand-new American Society for Microbiology journal dedicated to publishing interdisciplinary work at the interfaces of (micro)biological levels of organization. I had just taken a tenure-track assistant professorship and was in the middle of launching my independent research program in microbiome resilience. While intrigued by the opportunity, I was initially hesitant about how to respond: would serving a new journal be too demanding or too risky for an assistant professor who was supposed to be focused on establishing her research? I turned to my colleague Jim Tiedje for advice. Jim shared the story of his early career involvement with a different ASM journal, *Applied Microbiology*, which evolved into *Applied and Environmental Microbiology* (AEM) after he advocated for the name update to welcome research from the rapidly expanding frontier of environmental microbial ecology. I was inspired by Jack’s bold vision for *mSystems* and Jim’s sage insights that clarified the unique opportunity I faced. This would be a chance to contribute to shaping the future of our research. We could innovate new directions, blur disciplinary boundaries, and advance the goals of microbial systems research on behalf of the community. I accepted. In 2022, I “graduated” to become a senior editor.

### Systems research is a way of doing science

It is an approach, even a philosophy, that intentionally considers the interacting levels of biological organization as a unit while not losing sight of their individual contributions. Systems microbiology offers an expansive scientific vantage point, approaches, and technologies that are arguably necessary to address the complicated urgent challenges facing humanity today. Due to their complexity, these challenges simultaneously require a panoramic view and an embrace of details, contingencies, and contexts. What are some examples of challenges that demand the approach of systems microbiology?

- Fighting climate change by leveraging microbes and their functions to support resilient ecosystems.
- Managing and shaping the host-microbiome for improved human, other animal, and plant health.
- Ensuring the holistic well-being of connected environmental and human systems, for example, by predicting and preventing the spread of antimicrobial resistance genes, contaminants, and pathogens among them.
- Building new biotechnological systems, from molecular pathways to microbial communities to synthetic ecosystems, that can provide new insights into emergent properties and perform critical jobs like product synthesis or bioremediation.
- Understanding the consequences of human societal systems for microbiome functions, co-evolution, and feedback.

And so many more than I could mention! I believe that systems microbiology approaches will push research to meet our challenges. *mSystems* will launch several new subject areas to reflect the latest progress in these and other emerging areas in systems microbiology. Furthermore, as the only ASM “mJournal” that publishes methods and protocols, *mSystems* will continue to spearhead access to leading-edge technologies, computational models, and quantitative tools that allow systems-level insights and make discoveries possible.

We will carry on *mSystems’s* legacy of innovation and experimentation. We will trial new initiatives to enable “more perfect” peer review and ensure a valuable and constructive author experience. I apply data-driven decision-making. Thus, we will assess new initiatives in the same manner as one would consider experimental data testing a hypothesis. Additionally, I will engage our community in the co-creation of these initiatives, so please look out for numerous opportunities to promote your ideas, provide feedback, and make an impact.

Especially today, it seems that scientists face heightened scrutiny around their work and motivations. We want *mSystems* to remain a trusted journal recognized for its scientific integrity, which includes a commitment to rigorous practices regarding data and code availability. Rich, multidimensional data types are often a hallmark of *mSystems* research papers, and we will strive to ensure that the data, code, and analysis tools uphold the highest community standards in terms of findability, transparency, and reproducibility.

Importantly, I want to celebrate and expand our *mSystems* community. Jack has recruited and engaged a community that spans so many dimensions of differences and diversity. Our reviewers, authors, and editors hail from all over the globe and collectively represent a wealth of research disciplines and a richness of unique experiences. We celebrate this, value our collective insights and empathy strengthened by our differences, and encourage new voices and perspectives to join. I will do everything possible to ensure that peer review is fair and transparent, that decisions are consistent while also evolving with the journal’s priorities, and that as many microbiologists as possible can see themselves represented on our board. You are welcome at *mSystems*.

Finally, I wholeheartedly encourage early-career researchers to advance their careers and make a meaningful impact with *mSystems*. This was my journey, and I aim to pay it forward. If you are an early-career researcher with a passion for uncompromising integrity, innovation, and advancing critical and pressing research, you can find a home and mentorship at *mSystems*. I encourage you to engage as an author, reviewer, and editor and to take advantage of the professional development opportunities we offer. Contact me with your ideas, feedback, and questions. I am here to empower you to shape the future of microbial systems research.

